# Supplementary material for: Prevalence of viral hepatitis B in Ghana between 2015 and 2019: A systematic review and meta-analysis
Source: PLoS One. 2020 Jun 12;15(6):e0234348. doi: 10.1371/journal.pone.0234348 (PMC7292378; doi:10.1371/journal.pone.0234348)
Supplement: S1 File — (PDF) [file pone.0234348.s001.pdf]

Appendix 1 Detailed Database Search Strategies Used To Retrieve Articles

Embase Detailed Search Strategy

1. 'hepatitis b'/exp =99,195
2 'chronic hepatitis b'/exp =10,217
3. 'hepatitis b virus'/exp =53,255
4. 'hepatitis b surface antigen'/exp =36,924
5. 'hepatitis b(e) antigen'/exp =15,823
6. 'hepatitis b core antigen'/exp =4,358
7. 'hepatitis b antigen'/exp =53,411
8. Hbv =65,597
9. Hepb =553
10. Hbeag =14,212
11. 'ghana'/exp =10,786
12. 'ghanaian'/exp =601
13. 'human'/exp =21,161,782
14. #1 OR #2 OR #3 OR #4 OR #5 OR #6 OR #7 OR #8 OR #9 OR #10 =149,257
15. #11 OR #12 =11,100
16. #14 AND #15 =193
17. #13 AND #16 =179
18. #13 AND #16 AND [2015-2019]/py =96

Pubmed Detailed Search Strategies

(((((((((("hepatitis b"[MeSH Terms] OR "hepatitis b"[All Fields]) OR ("hepatitis b virus"[MeSH Terms] OR "hepatitis b virus"[All Fields])) OR ("hepatitis b, chronic"[MeSH Terms] OR "chronic hepatitis b"[All Fields])) OR ("hepatitis b surface antigens"[MeSH Terms] OR "hepatitis b surface antigens"[All Fields] OR "hepatitis b surface antigen"[All Fields])) OR ("hepatitis b e antigens"[MeSH Terms] OR "hepatitis b e antigens"[All Fields] OR "hepatitis b e antigen"[All Fields])) OR ("hepatitis b core antigens"[MeSH Terms] OR "hepatitis b core antigens"[All Fields] OR "hepatitis b core antigen"[All Fields])) OR ("hepatitis b antigens"[MeSH Terms] OR "hepatitis b antigens"[All Fields] OR "hepatitis b antigen"[All Fields])) OR ("gamma-hydroxy-gamma-ethyl-gamma-phenylbutyramide"[Supplementary Concept] OR "gamma-hydroxy-gamma-ethyl-gamma-phenylbutyramide"[All Fields] OR "hepb"[All Fields])) OR hbv[All Fields]) OR ("hepatitis b e antigens"[MeSH Terms] OR "hepatitis b e antigens"[All Fields] OR "hbeag"[All Fields])) AND ("ghana"[MeSH Terms] OR "ghana"[All Fields])) AND ("humans"[MeSH Terms] OR "humans"[All Fields] OR "human"[All Fields]) AND ("2015/01/01"[PDAT] : "2019/09/30"[PDAT])
