# Supplementary material for: Prevalence of viral hepatitis B in Ghana between 2015 and 2019: A systematic review and meta-analysis
Source: PLoS One. 2020 Jun 12;15(6):e0234348. doi: 10.1371/journal.pone.0234348 (PMC7292378; doi:10.1371/journal.pone.0234348)
Supplement: S2 File — (PDF) [file pone.0234348.s002.pdf]

Newcastle-Ottawa Scale adapted for cross-sectional studies

Selection:

1. Representativeness of the sample:
    a. Truly representative of the average in the target population. * (all subjects or random sampling)
    b. Somewhat representative of the average in the target group. * (non-random sampling)
    c. Selected group of users/convenience sample.
    d. No description of the derivation of the included subjects.

2. Sample size:
    a. Justified and satisfactory (including sample size calculation). *
    b. Not justified.
    c. No information provided

3. Non-respondents:
    a. Proportion of target sample recruited attains pre-specified target or basic summary of non-respondent characteristics in sampling frame recorded. *
    b. Unsatisfactory recruitment rate, no summary data on non-respondents.
    c. No information provided

4. Ascertainment of the exposure (risk factor):
    a. Vaccine records/vaccine registry/clinic registers/hospital records only. **
    b. Parental or personal recall and vaccine/hospital records. *
    c. Parental/personal recall only.

Comparability: (Maximum 2 stars)

1. Comparability of subjects in different outcome groups on the basis of design or analysis. Confounding factors controlled.
    a. Data/ results adjusted for relevant predictors/risk factors/confounders e.g. age, sex, time since vaccination, etc. **
    b. Data/results not adjusted for all relevant confounders/risk factors/information not provided.

Outcome:

1. Assessment of outcome:
    a. Independent blind assessment using objective validated laboratory methods. **
    b. Unblinded assessment using objective validated laboratory methods. **
    c. Used non-standard or non-validated laboratory methods with gold standard. *
    d. No description/non-standard laboratory methods used.

2. Statistical test:
    a. Statistical test used to analyse the data clearly described, appropriate and measures of association presented including confidence intervals and probability level (p value). *
    b. Statistical test not appropriate, not described or incomplete.

Cross-sectional Studies:
Very Good Studies: 9-10 points

Newcastle-Ottawa Scale adapted for cross-sectional studies

Good Studies: 7-8 points
Satisfactory Studies: 5-6 points
Unsatisfactory Studies: 0 to 4 points

This scale has been adapted from the Newcastle-Ottawa Quality Assessment Scale for cohort studies to provide quality assessment of cross sectional studies[1].

[1] Herzog R, et al. Is Healthcare Workers' Intention to Vaccinate Related to their Knowledge, Beliefs and Attitudes? A Systematic Review. *BMC Public Health* 2013 **13**:154
