# Supplementary material for: Prevalence of viral hepatitis B in Ghana between 2015 and 2019: A systematic review and meta-analysis
Source: PLoS One. 2020 Jun 12;15(6):e0234348. doi: 10.1371/journal.pone.0234348 (PMC7292378; doi:10.1371/journal.pone.0234348)
Supplement: S1 Appendix — (PDF) [file pone.0234348.s003.pdf]

Random effects model

Study name		Statistics for each study				
		Event rate	Lower limit	Upper limit	Z-Value	p-Value
Apiung	2017	0.0005	0.0000	0.0341	-3.4986	0.0005
Dassah	2015	0.0190	0.0047	0.0733	-5.4914	0.0000
		0.0055	0.0002	0.1423	-2.9949	0.0027

Event rate and 95% CI

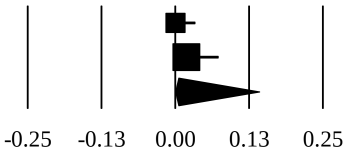

Test of Heterogeneity:[I2=60.84%, p=0.110]

S3 Appendix 3 Forest plot of HBV prevalence among pre-school children in Ghana
