# Supplementary material for: Prevalence of viral hepatitis B in Ghana between 2015 and 2019: A systematic review and meta-analysis
Source: PLoS One. 2020 Jun 12;15(6):e0234348. doi: 10.1371/journal.pone.0234348 (PMC7292378; doi:10.1371/journal.pone.0234348)
Supplement: S2 Appendix — (PDF) [file pone.0234348.s004.pdf]

Random effects model

Study name		Statistics for each study				
		Event rate	Lower limit	Upper limit	Z-Value	p-Value
Ampah	2016	0.0810	0.0675	0.0970	-24.1034	0.0000
De Mendoza	2019	0.0850	0.0585	0.1220	-11.5735	0.0000
		0.0818	0.0694	0.0961	-26.7370	0.0000

Event rate and 95% CI

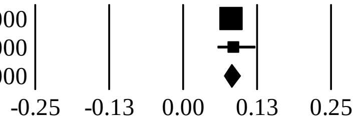

Test of Heterogeneity:[I2=0%, p=0.818]

S4 Appendix 4 Forest plot of HBV prevalence among the general population (community members and outpatients) in Ghana
