# Supplementary material for: Prevalence of viral hepatitis B in Ghana between 2015 and 2019: A systematic review and meta-analysis
Source: PLoS One. 2020 Jun 12;15(6):e0234348. doi: 10.1371/journal.pone.0234348 (PMC7292378; doi:10.1371/journal.pone.0234348)
Supplement: S3 Appendix — (PDF) [file pone.0234348.s005.pdf]

Random effects model

Study name		Event rate	Lower limit	Upper limit	Z-Value	p-Value
Lokpo	2017	0.0723	0.0677	0.0772	-70.6758	0.0000
Lokpo	2017	0.0694	0.0621	0.0775	-42.6525	0.0000
Osei	2017	0.0750	0.0561	0.0995	-15.8813	0.0000
		0.0717	0.0678	0.0757	-84.0597	0.0000

Statistics for each study

Event rate and 95% CI

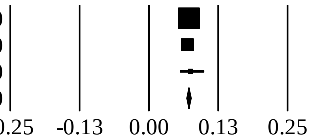

Test of Heterogeneity:[I2=0%, p=0.784]

S5 Appendix 5 Forest plot of HBV prevalence among blood donors in Ghana
