# Supplementary material for: Prevalence of viral hepatitis B in Ghana between 2015 and 2019: A systematic review and meta-analysis
Source: PLoS One. 2020 Jun 12;15(6):e0234348. doi: 10.1371/journal.pone.0234348 (PMC7292378; doi:10.1371/journal.pone.0234348)
Supplement: S4 Appendix — (PDF) [file pone.0234348.s006.pdf]

Random effects model

Study name		Event rate	Lower limit	Upper limit	Z-Value	p-Value
Archampong	2016	0.0830	0.0738	0.0932	-36.9475	0.0000
Boateng	2019	0.1250	0.0960	0.1612	-12.8710	0.0000
Pappoe	2019	0.0660	0.0453	0.0952	-13.0590	0.0000
Kye-Duodo	2016	0.0880	0.0615	0.1244	-11.8499	0.0000
		0.0892	0.0703	0.1125	-17.6372	0.0000

Statistics for each study

Event rate and 95% CI

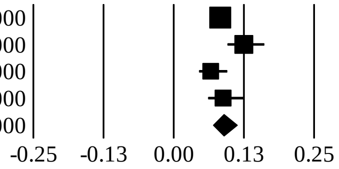

Test of Heterogeneity:[I2=69.91%, p=0.019]

S6 Appendix 6 Forest plot of HBV prevalence among HIV patients in Ghana
