# Supplementary material for: Prevalence of viral hepatitis B in Ghana between 2015 and 2019: A systematic review and meta-analysis
Source: PLoS One. 2020 Jun 12;15(6):e0234348. doi: 10.1371/journal.pone.0234348 (PMC7292378; doi:10.1371/journal.pone.0234348)
Supplement: S5 Appendix — (PDF) [file pone.0234348.s007.pdf]

Random effects model

Group by TEST	Study name	Event rate	Lower limit	Upper limit	Z-Value	p-Value
		Statistics for each study				
ELISA	Archampong 2016	0.0830	0.0738	0.0932	-36.9475	0.0000
ELISA	Kye-Duodo 2016	0.0880	0.0615	0.1244	-11.8499	0.0000
ELISA		0.0850	0.0573	0.1244	-10.9790	0.0000
RDT	Boateng 2019	0.1250	0.0960	0.1612	-12.8710	0.0000
RDT	Pappoe 2019	0.0660	0.0453	0.0952	-13.0590	0.0000
RDT		0.0938	0.0618	0.1400	-9.8306	0.0000
Overall		0.0890	0.0669	0.1175	-14.7331	0.0000

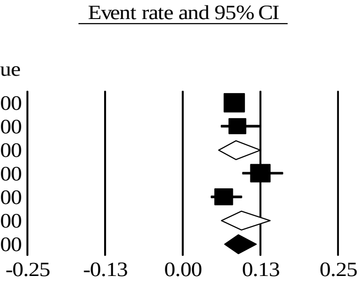

Test of Heterogeneity:[Overall: I2=69.91%, p=0.019, ELISA: I2=0%, p=0.758, RDT: I2=87.08%, p<0.005, Total between: p=0.732]

S7 Appendix 7 Forest plot of subgroup analysis of hepatitis B prevalence among HIV patients and by test type
