# Supplementary material for: Prevalence of viral hepatitis B in Ghana between 2015 and 2019: A systematic review and meta-analysis
Source: PLoS One. 2020 Jun 12;15(6):e0234348. doi: 10.1371/journal.pone.0234348 (PMC7292378; doi:10.1371/journal.pone.0234348)
Supplement: S6 Appendix — (PDF) [file pone.0234348.s008.pdf]

Random effects model

Study name		Statistics for each study				
		Event rate	Lower limit	Upper limit	Z-Value	p-Value
Helegbe	2018	0.0420	0.0355	0.0496	-35.0771	0.0000
Anabire	2019	0.0770	0.0663	0.0893	-30.1340	0.0000
Luuse	2016	0.0240	0.0100	0.0564	-8.1790	0.0000
Volker	2017	0.1670	0.1186	0.2299	-7.9065	0.0000
Ephraim	2015	0.0950	0.0590	0.1495	-8.5665	0.0000
Frempong	2019	0.1000	0.0547	0.1760	-6.5917	0.0000
		0.0744	0.0471	0.1155	-10.1769	0.0000

Event rate and 95% CI

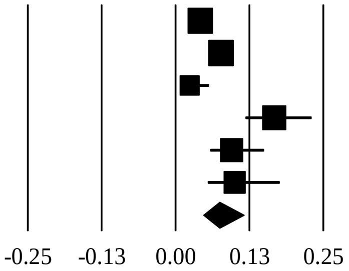

Test of Heterogeneity:[I2=92.69%, p<0.001]

S8 Appendix 8 Forest plot of HBV prevalence among pregnant women in Ghana
