# Supplementary material for: Prevalence of viral hepatitis B in Ghana between 2015 and 2019: A systematic review and meta-analysis
Source: PLoS One. 2020 Jun 12;15(6):e0234348. doi: 10.1371/journal.pone.0234348 (PMC7292378; doi:10.1371/journal.pone.0234348)
Supplement: S7 Appendix — (PDF) [file pone.0234348.s009.pdf]

Random effects model

Group by YEAR OF PUBLICATION	Study name		Event rate	Lower limit	Upper limit	Z-Value	p-Value
2015.00	Ephraim	2015	0.0950	0.0590	0.1495	-8.5665	0.0000
2015.00			0.0950	0.0590	0.1495	-8.5665	0.0000
2016.00	Luuse	2016	0.0240	0.0100	0.0564	-8.1790	0.0000
2016.00			0.0240	0.0100	0.0564	-8.1790	0.0000
2017.00	Volker	2017	0.1670	0.1186	0.2299	-7.9065	0.0000
2017.00			0.1670	0.1186	0.2299	-7.9065	0.0000
2018.00	Helegbe	2018	0.0420	0.0355	0.0496	-35.0771	0.0000
2018.00			0.0420	0.0355	0.0496	-35.0771	0.0000
2019.00	Anabire	2019	0.0770	0.0663	0.0893	-30.1340	0.0000
2019.00	Frempong	2019	0.1000	0.0547	0.1760	-6.5917	0.0000
2019.00			0.0782	0.0676	0.0903	-30.8352	0.0000
Overall			0.0649	0.0586	0.0718	-48.1272	0.0000

Statistics for each study

Event rate and 95% CI

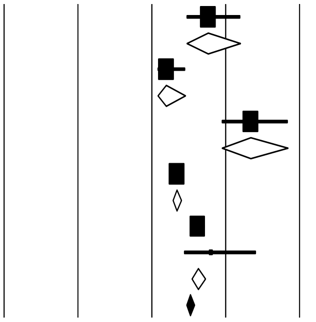

Test of Heterogeneity:[Overall: I2=92.67%, p<0.001, 2015-2018: I2=0%, p=1.000, 2019: I2=0%, p<0.404, Total between: p=0.001]

S9 Appendix 9 Forest plot of hepatitis B prevalence subgroup analysis among pregnant women by study publication year
