# Supplementary material for: Prevalence of viral hepatitis B in Ghana between 2015 and 2019: A systematic review and meta-analysis
Source: PLoS One. 2020 Jun 12;15(6):e0234348. doi: 10.1371/journal.pone.0234348 (PMC7292378; doi:10.1371/journal.pone.0234348)
Supplement: S8 Appendix — (PDF) [file pone.0234348.s010.pdf]

Random effects model

Study name		Statistics for each study				
		Event rate	Lower limit	Upper limit	Z-Value	p-Value
Adoba	2015	0.1450	0.1027	0.2009	-8.8354	0.0000
Adjei	2016	0.1420	0.0875	0.2221	-6.4642	0.0000
		0.1440	0.1089	0.1879	-10.9474	0.0000

Event rate and 95% CI

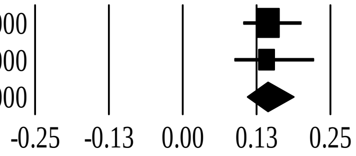

Test of Heterogeneity:[I2=0%, p=0.943]

S10 Appendix 10 Forest plot of HBV prevalence among special occupation population (Barbers and long distance drivers) in Ghana
